# Feasibility of an Electronic Patient-Reported Outcome System in People Living With HIV: Retrospective Analysis of a Mobile App-Based Pilot Study

**DOI:** 10.2196/71493

**Published:** 2025-06-10

**Authors:** Yusuke Yoshino, Yoshitaka Kimura, Yoshitaka Wakabayashi, Takatoshi Kitazawa

**Affiliations:** 1 Department of Microbiology and Immunology, School of Medicine, Teikyo University, 2-11-1, Kaga, Itabashi, Tokyo, 1738605, Japan, 81-3-3964-1211, 81-3-3964-1211; 2 Infectious Disease Group, Internal Medicine, Teikyo University Hospital, Tokyo, Japan; 3 Asia International Institute of Infectious Disease Control, Teikyo University, Tokyo, Japan

**Keywords:** patient reported outcome, smartphone app, quality of life, feasibility test, electronic patient reported outcome

## Abstract

**Background:**

Advances in antiretroviral therapy have transformed HIV into a manageable chronic condition, allowing people living with HIV to live longer. However, with aging, people living with HIV face increased risks of lifestyle-related diseases and unaddressed psychosocial issues, including stigma, discrimination, and mental health concerns. Patient-reported outcomes are essential tools in person-centered care and have demonstrated clinical utility in oncology and rheumatology; yet remains underutilized in infectious disease settings. Recently, the European AIDS Clinical Society has recommended electronic patient-reported outcome (ePRO) systems to support HIV care.

**Objective:**

This study aimed to evaluate the feasibility and clinical utility of a smartphone-based ePRO system specifically developed for people living with HIV in Japan.

**Methods:**

A retrospective study was conducted among people living with HIV who attended the HIV outpatient clinic at Teikyo University Hospital between July and September 2022. Participants who consented to use the ePRO system installed a smartphone app and completed the HIV symptom index prior to their clinic visit. Physicians reviewed the responses during consultations; following the visits, patients completed a usability survey addressing clarity, response time, satisfaction, communication quality, and intention for future use. Medical records were reviewed to determine any new symptoms, findings, or medical actions that had not been documented in the previous two years.

**Results:**

A total of 27 people living with HIV (median age, 46 years; 100% male) used the ePRO app, and 25 (93%) completed the postuse questionnaire. Of these, 19/25 (76%) completed the Symptom Index within 5 minutes, while one participant required more than 15 minutes. Regarding usability, 76% (n=19) reported being satisfied or very satisfied, and 76% (n=19) found the system useful in improving communication with their provider. Additionally, 76% (n=19) expressed willingness to use the system again, while 5 (20%) participants indicated interest only if improvements were made. Medical record analysis revealed that 17/27 patients (63%) had new clinical information documented, including mental health symptoms (n=7, 26%), skin problems (n=7, 26%), and new diagnoses or treatment changes in 6 (22%) cases. Over 40% (n=11) of patients reported issues such as anxiety, insomnia, dermatological symptoms, or concerns related to body image.

**Conclusions:**

This pilot study demonstrated that a smartphone-based ePRO system for people living with HIV is feasible and well-accepted in real-world clinical practice. It facilitated early detection of psychosocial and physical issues that may otherwise be overlooked in routine care and improved patient–provider communication. These findings support the integration of ePRO systems into HIV care and underscore the need for further refinement of the app and prospective studies to assess long-term impact on patient outcomes and quality of care.

## Introduction

Advances in HIV treatment have transformed it from incurable to a manageable disease, requiring continuous use of medication to suppress viral growth. However, as people living with HIV live longer, the risk of lifestyle diseases such as diabetes increases with age [1]. Additionally, previous studies have shown that stigma and discrimination remain prevalent, potentially leading to psychological issues [2]. This has increased the demand for patient-centered medicine beyond viral suppression.

Patient-reported outcome (PROs) are gaining attention as tools for patient-centered care. PROs are based on patients’ direct responses to questionnaires. Their validity has been evaluated in various studies, and PROs can assess symptoms, functioning, health status, and quality of life (QOL) [3]. PROs can be disease-specific or general, applicable across conditions. Clinicians can select appropriate PROs for care and research.

PROs are increasingly used for diseases affecting long-term QOL, including malignancies. Studies have revealed gaps between patients and providers in assessing treatment side-effects. Patient-centered evaluations are crucial for improving health-related QOL (HRQOL) in cancer care [4]. In collagen diseases, PROs are used to evaluate symptoms like pain and improve care quality and HRQOL. Thus, PROs are valuable for enhancing care and supporting patient-centered medicine.

PROs have rarely been evaluated in infectious diseases, particularly acute infections, which often resolve with short-term treatment. We previously assessed PROs to measure QOL in patients with community-acquired influenza [5]. After antiviral treatment for influenza, mental QOL worsened during the course. Physical QOL was higher in vaccinated than in nonvaccinated patients [6]. These findings suggest PROs may benefit HIV care, as HIV has become a chronic disease.

In Europe and the United States, PROs are used in HIV trials to evaluate efficacy and adverse effects. Experts report that PROs improve HRQOL in people living with HIV when applied in practice. Recently, an electronic patient-reported outcome (ePRO) system was developed for real-time health assessment via tablets or computers in clinics. The European AIDS Clinical Society 2023 guidelines recommend this ePRO system [7]. In 2022, we collaborated with Integrity Healthcare Corporation and Gilead Sciences Inc. to develop a home-based mobile ePRO app. Here, we present a feasibility study of our prototype mobile ePRO app and its utility.

## Methods

### Study Design and Setting

The responses of a postuse questionnaire and the impact of the ePROs on clinical practice were retrospectively evaluated among outpatients who visited the Teikyo University Hospital, Tokyo, Japan, between July and September 2022. Participants had agreed to use the ePRO app, particularly during their clinic visits. First, the participants were asked to install the newly developed mobile app on their phones. Informed consent to use the app was obtained from all participants. They responded to the PRO through the app during the week preceding their clinic visit. During the visit, the clinician reviewed the PRO results and treated the participants. All people living with HIV visiting the clinic during the observation period were introduced to the app, and those who agreed to use it on the same day were included in the analysis.

After the clinic visit, the PROs answered via the app were evaluated through a survey of questions concerning the clarity of content, time required to answer, satisfaction with use, changes in quality of communication with the clinician, and desire to use PROs in the future. Satisfaction was assessed with the following question, rated on a 5-point scale (very satisfied, satisfied, neutral, somewhat dissatisfied, dissatisfied): “How was your experience with the consultation after answering the HIV-SI via the app and sharing the results with your doctor in advance?” In addition, medical records from the consultation when the PRO was used were reviewed to identify any new symptoms or findings that had not existed in the past two years, or if any additional medical procedures initiated. The ePRO results were also summarized.

The ePRO content was based on the HIV symptom index [8], comprising 20 items, including those on physical findings, psychological problems, sexual activity, and body image. Each item was rated on a five-point scale: (1) I did not have this symptom, (2) I was not bothered, (3) I was a little bothered, (4) I was indeed bothered, and (5) I was bothered a lot. Responses to the questions were available to the responsible physician immediately after the people living with HIV submitted them online.

The number of participants (n) and corresponding percentages (%) are reported in this paper.

### Ethical Considerations

This study was conducted in accordance with the Declaration of Helsinki and approved by the Teikyo University Ethics Committee (approval number: 23‐009). Due to the retrospective nature of the study and the use of anonymized data, the requirement for written informed consent was waived. Instead, an opt-out method was used to inform patients about the study via the institution’s website, providing them with the opportunity to decline participation. All data were fully anonymized prior to analysis, and no identifying information was included. No financial compensation or other incentives were provided to participants.

## Results

Twenty-seven patients used the mobile app in clinical practice. All patients were male, with a median age of 46 years. All were taking antiretroviral treatment, had maintained a CD4-positive lymphocyte count >200/μL for the past six months, and had a viral load <200 copies.

Twenty-five patients returned the postuse questionnaire. Nineteen of 25 (76%) respondents reported it took 5 minutes to complete; 5 (20%) reported 5-10 minutes, and one (4%) reported >15 minutes. Regarding satisfaction, 19 (76%) patients were very satisfied or satisfied, 5 (20%) were neutral, and one (4%) was somewhat dissatisfied. Nineteen (76%) patients found the questionnaire very or mostly useful for communication; 6 (24%) patients reported it to be neutral. Regarding future use, 19 (76%) patients wished to continue, 5 (20%) preferred use with improvements, and 1 did not.

Medical record review showed that 17/27 (63%) patients had additional information not recorded in the past two years. Of these, 7 (26%) reported mental health–related symptoms and 7 (26%) had skin problems. In 6 (22%) patients, PRO usage led to new treatments or actions not taken in the previous two years. Three patients were referred to dermatology, and 1 was newly diagnosed with chronic obstructive pulmonary disease following a complaint of respiratory symptoms.

Over 40% (n=11) patients reported symptoms including anxiety, sleep issues, rashes, dry or itchy skin, and appearance changes from weight or fat gain.

## Discussion

This study demonstrated the feasibility and clinical utility of a newly developed ePRO smartphone app for people living with HIV. The system was well accepted, with most users completing the questionnaire in under 5 minutes and reporting high satisfaction. The ePRO facilitated improved communication between patients and clinicians and led to documentation of new clinical information in nearly two-thirds of participants. These findings support the hypothesis that an ePRO system tailored for people living with HIV can enhance patient-centered care and identify unrecognized issues.

The short completion time and favorable user responses suggest that the app’s design featuring fewer questions and a simplified interface, contributed to its high acceptability. This aligns with studies emphasizing minimal patient burden when implementing PROs in chronic disease care [3]. The enhanced documentation of mental and dermatological symptoms highlights how ePROs can uncover patient concerns that may be under-reported during standard consultations. These findings align with literature showing PROs improve HRQOL by better capturing subjective symptoms [9].

Notably, mental health issues common among people living with HIV often go undiagnosed due to their subjective nature and limited psychiatric training among providers. Similarly, skin-related concerns such as rashes or appearance changes are frequently under-recognized in routine care. ePROs appear to bridge this gap by allowing patients to express symptoms that may not surface during clinician-led interviews. Compared to paper- or tablet-based PROs, smartphone apps offer greater accessibility and real-time symptom reporting from any location. As smartphone use increases globally, especially among younger generations, app-based ePROs hold promise for broader implementation in HIV care and beyond.

This study has limitations. First, the small sample from a single center may limit generalizability. Second, the retrospective design and lack of a control group prevent causal inference. Third, only the HIV symptom index was used; the utility of other PRO tools remains unexplored. While most participants expressed satisfaction, some suggested improvements, indicating room for optimization. Finally, the short observation period limits assessment of long-term utility and sustainability.

The findings of this study underscore the feasibility and clinical relevance of incorporating smartphone-based ePRO systems into HIV outpatient care. By enabling the identification of otherwise undocumented symptoms and improving communication between patients and providers, ePROs may contribute to more holistic and person-centered care models. These findings support the integration of digital health tools in HIV management and highlight the potential for ePROs to address unmet needs in clinical communication and symptom assessment. Future research should refine the app, broaden use, and evaluate long-term outcomes of routine ePRO implementation.

## abbreviations

ePRO: electronic PRO
HRQOL: health-related quality of life
PRO: patient-reported outcome
QOL: quality of life
